# Peptidyl-Prolyl *Cis/Trans* Isomerase Pin1 and Alzheimer’s Disease

**DOI:** 10.3389/fcell.2020.00355

**Published:** 2020-05-15

**Authors:** Long Wang, Ying Zhou, Dongmei Chen, Tae Ho Lee

**Affiliations:** ^1^Fujian Key Laboratory for Translational Research in Cancer and Neurodegenerative Diseases, Institute for Translational Medicine, School of Basic Medical Sciences, Fujian Medical University, Fuzhou, China; ^2^Key Laboratory of Brain Aging and Neurodegenerative Diseases of Fujian Provincial Universities and Colleges, School of Basic Medical Sciences, Fujian Medical University, Fuzhou, China

**Keywords:** Alzheimer’s disease, amyloid precursor protein (APP), Pin1, phosphorylation, tau

## Abstract

Alzheimer’s disease (AD) is the most common cause of dementia with cognitive decline. The neuropathology of AD is characterized by intracellular aggregation of neurofibrillary tangles consisting of hyperphosphorylated tau and extracellular deposition of senile plaques composed of beta-amyloid peptides derived from amyloid precursor protein (APP). The peptidyl-prolyl *cis/trans* isomerase Pin1 binds to phosphorylated serine or threonine residues preceding proline and regulates the biological functions of its substrates. Although Pin1 is tightly regulated under physiological conditions, Pin1 deregulation in the brain contributes to the development of neurodegenerative diseases, including AD. In this review, we discuss the expression and regulatory mechanisms of Pin1 in AD. We also focus on the molecular mechanisms by which Pin1 controls two major proteins, tau and APP, after phosphorylation and their signaling cascades. Moreover, the major impact of Pin1 deregulation on the progression of AD in animal models is discussed. This information will lead to a better understanding of Pin1 signaling pathways in the brain and may provide therapeutic options for the treatment of AD.

## Introduction

Alzheimer’s disease (AD) is the most common form of dementia, accounting for 50–75% of all cases, and presents as a series of cognitive or behavioral symptoms including decline in memory (Mckhann et al., 2011; Lane et al., 2018; Alzheimer’s Association, 2019). The progression of AD may drive or be exacerbated by various systemic abnormalities, such as abnormalities in systemic immunity, metabolic disorders, cardiovascular disease, and sleep disorders (Wang et al., 2017). Approximately 50 million people worldwide currently suffer from dementia, and this number is expected to triple in the next three decades due to the increasing number of aging people (Lane et al., 2018). The neuropathological hallmarks of AD are the intracellular aggregation of neurofibrillary tangles (NFTs) containing paired helical filaments (PHFs) consisting of hyperphosphorylated tau protein and the extracellular deposition of senile plaques (SPs) composed of beta-amyloid (Aβ) peptides derived from amyloid precursor protein (APP) (Hardy and Selkoe, 2002; Binder et al., 2005; Goedert and Spillantini, 2006; Roberson and Mucke, 2006; Ballatore et al., 2007; Ittner and Gotz, 2011). However, the molecular link and mechanisms underlying the pathogenesis of AD are not fully understood. Therefore, understanding the early disease mechanisms responsible for neurodegeneration in AD is critical for identifying proper diagnostic approaches and new effective therapeutic targets.

Protein phosphorylation is one of the major post-translational modifications and is involved in diverse cellular processes regulating numerous physiological and pathological processes (Cohen, 1982; Nestler and Greengard, 1983; Oliveira et al., 2017; Butterfield, 2019). In particular, serine or threonine residues preceding proline (S/T-P) are the most frequently phosphorylated motifs in AD (Lu et al., 2002, 2003; Lu, 2004; Lu and Zhou, 2007; Iqbal et al., 2016). Interestingly, due to its unique five-carbonyl ring structure, proline is able to present as two strikingly distinct conformations, *cis* and *trans* (Lu et al., 1996; Ranganathan et al., 1997; Yaffe et al., 1997; Wulf et al., 2005; Lu et al., 2007; Lu and Zhou, 2007). The peptide bond dihedral angle ω of proline residue adopts either about 0° (*cis* conformation) or about 180° (*trans* conformation), which plays critical roles in the rate-determining steps of protein folding, thus controlling the biological activity of proteins and their cellular progression (Wedemeyer et al., 2002; Andreotti, 2003; Fischer and Aumuller, 2003; Cortes-Hernandez and Dominguez-Ramirez, 2017). The spontaneous interconversion of *cis*/*trans* isomerization occurs slowly but can be catalyzed by a number of peptidyl-prolyl *cis*/*trans* isomerases (PPIases), such as cyclophilins, FK506-binding proteins (FKBPs), and parvulin-type PPIases (Fischer and Aumuller, 2003; Lu and Zhou, 2007). Cyclophilins and FKBPs not only belong to immunophilins which are cellular targets for the immunosuppressive drugs, but also have relationships with tau-related and Aβ pathology (Blair et al., 2015). Cyclophilin D is one of the most unique and well-studied cyclophilins, and cyclophilin D deficiency can protect neurons from Aβ- and oxidative stress-induced toxicity (Du et al., 2008, 2014; Guo et al., 2013). FKBP with a molecular mass of ∼52 kDa (FKBP52) is one of the most well-studied FKBPs, and FKBP52 has been shown to be highly expressed in neurons and abnormally low in AD brains (Giustiniani et al., 2012, 2014, 2015). Nevertheless, the phosphorylation of an S/T-P motif further slows the spontaneous isomerization rate and renders the peptide bond against the catalytic action of known PPIases (Wulf et al., 2005; Lu and Zhou, 2007). Thus, the important discovery of Pin1 has shed light on the significance of this intrinsic conformational switch in human physiology and pathology.

Pin1 (protein interacting with NIMA (never in mitosis A)-1) was originally identified in a yeast genetic and biochemical screen for proteins involved in mitotic regulation (Lu et al., 1996, 2002). The yeast Pin1 homolog Ess1 has been found to be the only enzyme being essential for survival among 13 PPIases since its discovery (Hanes et al., 1989; Lu, 2004). The human Pin1 has 163 amino acids with a molecular mass of 18 kDa, containing an N-terminal WW domain (residues 1–39) characterized by two invariant tryptophans and a C-terminal PPIase domain (residues 50–163) which shares little similarity with cyclophilins and FKBPs (Lu et al., 1996; Ranganathan et al., 1997). Pin1 is a unique and conserved PPIase that binds to specific phosphorylated proline-directed serine or threonine (pS/T-P) motifs and catalyzes the *cis/trans* isomerization of peptidyl-prolyl peptide bonds (Lu et al., 1996, 1999b; Ranganathan et al., 1997; Yaffe et al., 1997; Schutkowski et al., 1998; Shen et al., 1998). The unique substrate specificity of Pin1 results from the organization of active site residues (Ranganathan et al., 1997; Lu et al., 2002). Specifically, the residues L122, M130, and F134 form a hydrophobic binding pocket for the substrate proline, and the cluster sequestering K63, R68, and R69 forms a positive charged phosphate binding loop which either interacts with a bound sulfate ion or facilitates binding to the pS/T-P motif (Ranganathan et al., 1997; Behrsin et al., 2007; Lee and Liou, 2018). Further studies revealed that mutation of R68 and R69 could abolish the striking phosphorylation-specificity completely but barely affect the basic enzymatic activity (Yaffe et al., 1997; Zhou et al., 2000; Lu et al., 2002). In addition, the WW domain has been shown to target Pin1 to the substrates since it has a higher affinity to phosphorylated peptides as compared to the PPIase domain (Lu et al., 1999b; Smet et al., 2005). This Pin1-mediated conformational change of its substrates regulates numerous cellular processes, such as cell-cycle progression, cellular stress responses, development, neuronal function, immune responses, and cell death (Zhou et al., 1999; Lu and Zhou, 2007). Notably, Pin1 deregulation is implicated in age-dependent human diseases, including cancer and AD (Lu and Zhou, 2007; Lee et al., 2011b; Zhou and Lu, 2016). Pin1 activity and expression are significantly inhibited in human AD brains and highly increased in diverse types of cancers, indicating that Pin1 might have important roles in both proliferation and degeneration (Lu et al., 1999a; Liou et al., 2003; Butterfield et al., 2006; Lu and Zhou, 2007; Lee et al., 2011b; Driver et al., 2012; Zhou and Lu, 2016; Chen et al., 2020).

This review focuses on the deregulation of Pin1 in AD brains, the currently understood mechanisms of tau hyperphosphorylation and APP processing associated with Pin1, and the major impact of Pin1 deregulation on AD development. This advanced understanding of the involvement of the Pin1 signaling pathway in phosphorylation will support Pin1 as a novel potential diagnostic and therapeutic target.

## Regulation of Pin1 in AD

### Pin1 Expression in AD

The significantly different levels of soluble and functional Pin1 between the brain samples of patients with AD and the control brain samples from age-matched normal subjects suggest a possible protective role of Pin1 against AD. A large amount of soluble Pin1 is dramatically depleted and sequestrated in NFTs in the human AD brain but not in age-matched normal brains (Lu et al., 1999a). Pin1 expression has been further examined in the human hippocampus, a brain region that is particularly vulnerable to AD damage at early stages (Liou et al., 2003; Mu and Gage, 2011). In the hippocampus of normally aged brain samples, the expression of Pin1 in the CA1 region and subiculum is relatively lower than that in the CA4, CA3, and CA2 regions and presubiculum (Liou et al., 2003; Lu et al., 2003). Notably, in AD brains, NFTs predominantly occur in the CA1 region and subiculum, consistent with the finding that these subregions are prone to pyramidal neuron loss in AD (Davies et al., 1992; Liou et al., 2003). Indeed, among a randomly selected pool of 1,000 pyramidal neurons in AD, 96% of pyramidal neurons with higher expression of Pin1 seem to avoid tau-related pathology, while 71% of neurons with lower expression of Pin1 are vulnerable to NFT formation (Liou et al., 2003). On the contrary, some groups also reported that Pin1 was localized to granular vesicles but not to tau aggregates in AD (Holzer et al., 2002; Ramakrishnan et al., 2003; Dakson et al., 2011; Ando et al., 2013). Recently, according to the hippocampal gene expression profiles of patients from three distinct age groups, the expression of Pin1 is decreased slightly in the aging group but is dramatically decreased in the AD group compared with the young group (Lanke et al., 2018). These results suggest that reduced expression of Pin1 may contribute to the development of AD, including neurofibrillary degeneration.

### Pin1 Genetics in AD

The apolipoprotein E (APOE) ε4 allele was the first definitive gene to be implicated in late-onset AD (LOAD) and is located on chromosome 19q13.2 (Corder et al., 1993; Huq et al., 2019; Yamazaki et al., 2019). Although the human Pin1 gene is located on the same chromosome, this locus has been identified as a novel LOAD locus and is independent of APOE (Wijsman et al., 2004). Currently, three single nucleotide polymorphisms (SNPs) in the promoter region of the Pin1 gene have been identified to investigate their correlations with AD, including rs2287839 (-5185 G/C), rs2233678 (-842 G/C), and rs2233679 (-667 T/C). All of Pin1 polymorphism studies were conducted using genomic DNA from blood cells between AD patients and age-matched normal subjects. The polymorphism rs2233678 results in decreased Pin1 levels and is associated with a significantly raised risk of developing AD (Segat et al., 2007). The polymorphism rs2287839 leads to increased Pin1 expression and is correlated with 3-year delayed onset of LOAD (Ma et al., 2012b). However, other groups showed that polymorphisms in the promoter of Pin1, rs2233678 and rs2233679, were not associated with increased LOAD risk (Lambert et al., 2006; Nowotny et al., 2007; Cao et al., 2013). Interestingly, rs2233678 and rs2233679 have also been shown to decrease Pin1 expression and are implicated in the decreased risk of breast cancer, lung cancer, and nasopharyngeal carcinoma (Han et al., 2010; Lu et al., 2011, 2013). Therefore, since the controversial results remain to be elucidated, further validation of large prospective studies is needed to verify the roles of Pin1 polymorphisms in AD. Recently, a highly pathogenic and novel somatic single nucleotide variation (SNV) in Pin1 has been found in the hippocampal formation (HIF) of an AD patient (Park et al., 2019). Since the T152M mutation is located in the C-terminal PPIase domain of Pin1, the mutation might attenuate the enzymatic activity of Pin1 and increase tau hyperphosphorylation (Park et al., 2019). However, the molecular mechanism by which the somatic mutation regulates Pin1 activity and whether T152M knockin mice show tau-related and Aβ pathology remain to be elucidated.

### Pin1 Post-translational Modification in AD

Pin1 activity is regulated by post-translational modifications, including oxidation and phosphorylation, in AD. Neurons in the human brain are vulnerable to oxidative stress, and increased oxidative damage has been shown to be an early event in AD (Markesbery, 1997; Nunomura et al., 2001; Halliwell, 2006). Notably, Pin1 is modified by oxidation, leading to the loss of its activity in the hippocampus in AD (Butterfield et al., 2006; Sultana et al., 2006). Besides, oxidized Pin1 may be recognized by the ubiquitinylation system, giving rise to the polyubiquitination (Tramutola et al., 2018). By employing antibodies specifically recognizing oxidized C113 of Pin1, Pin1 oxidation on C113 has been identified to inactivate the catalytic activity of Pin1, and C113-oxidized Pin1 is elevated in human AD brains compared with age-matched controls (Chen et al., 2015). It is possible that an increased percentage of C113-oxidized Pin1 in response to oxidative stress may result in the inhibition of enzymatic activity and reduction of Pin1 levels. The loss of Pin1 activity induced by oxidative stress may also result in the loss of synaptic plasticity, which is the structural basis for memory impairment in AD (Xu et al., 2017). These results suggest that the protective roles of Pin1 may be attenuated by a variety of reactive oxygen species, which are common in human AD brains.

Recently, death-associated protein kinase 1 (DAPK1) has been found to play essential roles in neuronal cell death and various neurodegenerative diseases, including AD (Chen et al., 2019; Kim et al., 2019). Importantly, DAPK1 is capable of phosphorylating Pin1 at S71 in the PPIase domain, thus inhibiting its nuclear localization, prolyl isomerase activity, and cellular function (Lee et al., 2011a, b). DAPK1 dramatically increases tau protein stability and hyperphosphorylation at multiple AD-related sites, which is mediated by the inhibition of Pin1 activity by phosphorylation (Kim et al., 2014). DAPK1 phosphorylates and activates N-myc downstream-regulated gene 2 (NDRG2), resulting in increased tau phosphorylation via a reduction in Pin1 expression (Rong et al., 2017; You et al., 2017). In summary, the existence of Pin1 in the normal brain may have certain protective functions against AD, as decreased expression or declined activity of Pin1 make neurons vulnerable to pathologies related to AD.

## Pin1 and Tau-Related Pathology

The intracellular aggregation of NFTs containing PHFs made of hyperphosphorylated tau is one of the neuropathological hallmarks of AD (Geschwind, 2003; Binder et al., 2005; Goedert and Spillantini, 2006; Roberson and Mucke, 2006; Ballatore et al., 2007). Compared with Aβ pathology, which may play a critical role in AD pathogenesis, the prevalence of NFTs has a strong correlation with the severity of cognitive impairment, indicating that tau-related pathology may indicate the status of cognitive deficits and dementia (Nelson et al., 2012). Encoded by a single gene, MAPT, located on human chromosome 17, tau is a type of microtubule-associated protein and is expressed predominantly in the brain (Lee et al., 1989; Albayram et al., 2016; Iqbal et al., 2016). It is well-established that the physiological function of tau is to maintain microtubule-related functions, such as microtubule assembly and axonal transportation, and the abnormal hyperphosphorylation of tau inhibits normal microtubule functions and alters tau protein stability (Drubin and Kirschner, 1986; Bramblett et al., 1993; Alonso et al., 1994; Petrucelli et al., 2004; Shimura et al., 2004; Stoothoff and Johnson, 2005; Poppek et al., 2006). Specifically, abnormally phosphorylated tau is detached from microtubules and disrupts microtubule integrity (Iqbal et al., 2009, 2016). Hyperphosphorylated tau, but not normal tau, is a component of PHF-forming insoluble aggregates and further becomes NFTs (Lee et al., 1991; Goedert et al., 1992; Matsuo et al., 1994). These results indicate that the phosphorylation of tau is essential for the development of tau-related pathology.

**FIGURE 1:**
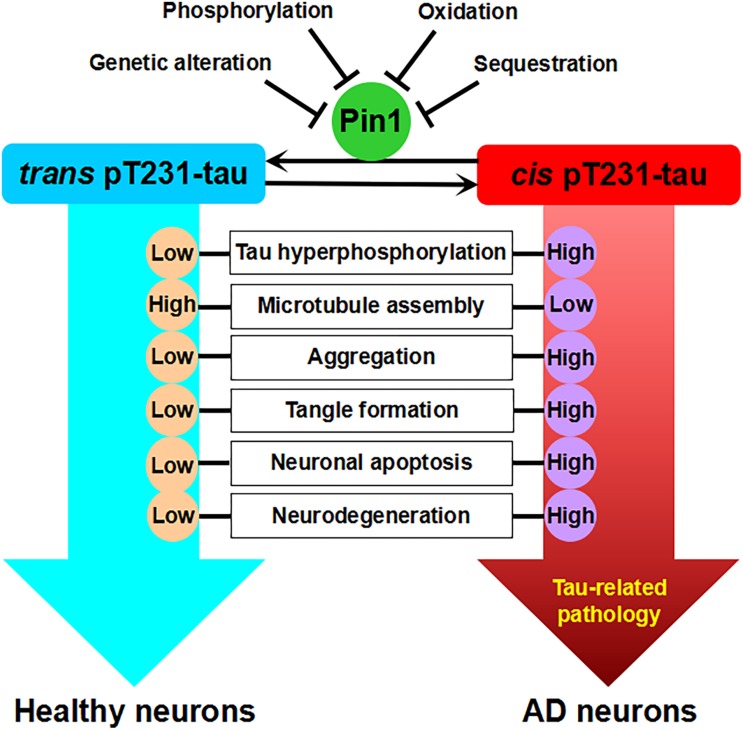
Pin1-regulated isomerization of pT231-tau against tau-related pathology. There are two strikingly distinct *cis* and *trans* conformations of the pT231 motif of tau after phosphorylation. *Cis*, but not *trans*, pT231-tau promotes tau hyperphosphorylation, the disruption of microtubule assembly, tau aggregation, tangle formation, neuronal apoptosis, and neurodegeneration. Pin1 binds to the pT231-P motif and isomerizes the *cis* form of pT231 to the *trans* form. Low levels of Pin1 due to genetic alteration or the inhibition of Pin1 expression or activity by phosphorylation, oxidation, and sequestration increase the levels of *cis* pT231-tau and may contribute to AD.

The phosphorylation of T231 (pT231), among a number of tau phosphorylation sites, appears to be the first detectable event during AD pretangle formation (Luna-Munoz et al., 2007). pT231 may play a critical role in regulating the conformation and misfolding process of tau (Lee et al., 2011b; Iqbal et al., 2016). Notably, Pin1 colocalizes with phosphorylated tau, directly binds to pT231-tau, and can restore its biological activity by promoting tau dephosphorylation to bind microtubules and increase microtubule assembly (Lu et al., 1999a, 2003; Ramakrishnan et al., 2003; Lu, 2004). Pin1 facilitates tau dephosphorylation through the proline-directed phosphatase PP2A, which has conformational specificity and dephosphorylates only the *trans* pS/T-P motif (Zhou et al., 2000). Pin1 has been found to bind PHFs and be trapped in tangles in the AD brain, resulting in the depletion of soluble Pin1 (Lu et al., 1999a). A recent *in vitro* study showed that reduced Pin1 expression led to the increase of pT231-tau levels (Park et al., 2019). Pin1 has been shown to accelerate the *cis* to *trans* isomerization of pT231-tau, restore its function, and maintain tau levels via proteasome-dependent proteolytic pathway (Poppek et al., 2006; Lim et al., 2008). However, Pin1 has no effect on T231A mutant tau (Lim et al., 2008; Nakamura et al., 2012). Interestingly, when hippocampal cultured neurons are exposed to Aβ42 oligomers, Pin1 can be activated to dephosphorylate pT231-tau mediated by PP2A (Bulbarelli et al., 2009). Notably, studies have showed that microtubule assembly can be significantly increased by unphosphorylated wild-type tau, but not phosphorylated tau which can be restored by PP2A, while the phosphorylated T231A tau is still able to promote microtubule assembly and this ability is not affected by Pin1, suggesting that T231 phosphorylation is critical for Pin1 to maintain microtubule function of tau (Nakamura et al., 2012). Therefore, tau hyperphosphorylation might induce tau aggregation which further sequestrates Pin1, thereby preventing pT231-tau dephosphorylation mediated by PP2A (Figure 1). However, other studies have questioned the specificity of Pin1 targeting site, as they revealed that Pin1 recognized other pS/T-P sites such as pT212 and pS235 motifs in full-length tau, which were the preferred substrates over pT231 motif (Smet et al., 2004, 2005; Landrieu et al., 2006; Kimura et al., 2013; Eichner et al., 2016). Besides, other studies also indicated that Pin1 did not regulate the microtubule function of phosphorylated tau (Lippens et al., 2007; Landrieu et al., 2010, 2011; Kutter et al., 2016; Lu et al., 2016; Rogals et al., 2016). Therefore, the specificity of Pin1 targeting sites of tau and the regulatory function of Pin1 toward phosphorylated tau raise other possibilities which need further investigation.

Recently, the Lu laboratory developed conformation-specific *cis* and *trans* polyclonal and monoclonal pT231-tau antibodies (Nakamura et al., 2012; Kondo et al., 2015). Specifically, *cis* pT231-tau appears to be more responsible for resistance to tau dephosphorylation and degradation, the disruption of microtubule structure, and vulnerability toward aggregation, and Pin1 catalyzes the isomerization of pT231-tau from *cis* to *trans*, restoring its ability to bind microtubules (Lu et al., 1999a, 2016; Nakamura et al., 2012; Albayram et al., 2016, 2018). Indeed, *cis* pT231-tau, but not *trans* pT231-tau, is significantly increased and localized to dystrophic neurites in human mild cognitive impairment (MCI) and AD brains (Nakamura et al., 2012). Furthermore, *cis* pT231-tau, but not *trans* pT231-tau, strongly correlates with neurofibrillary degeneration, which is associated with decreased Pin1 levels in the AD hippocampus, in accord with the binding of Pin1 to PHFs leading to the depletion of soluble Pin1 (Lu et al., 1999a; Nakamura et al., 2012). In addition, *cis* pT231-tau is dramatically induced, facilitates the disruption of axonal microtubules and organelle transport, and finally leads to neuronal apoptosis under neuronal stress (Kondo et al., 2015). Therefore, the neurotoxic *cis* pT231-tau may function as a critical driver of neurodegeneration, as it can spread among neurons in a prion-like fashion (Kondo et al., 2015; Albayram et al., 2018). In tau-overexpressing mice, while *trans* pT231-tau is barely detected in sarkosyl-insoluble fractions, *cis* pT231-tau levels are robustly increased in insoluble fractions in the brain (Nakamura et al., 2012). Interestingly, *cis* pT231-tau has been shown to be a major early driver of traumatic brain injury (Kondo et al., 2015; Albayram et al., 2017). These results suggest that the Pin1-regulated isomerization of the *cis* to *trans* conformations of phosphorylated tau is a key mechanism to protect against tau-related pathology. Nevertheless, the *cis* pT231-tau antibody raised against a peptide containing a chemically modified proline instead of a native *cis*-proline has also been questioned, and it is suggested that the specific pT231-P232 bond in phosphorylated tau be majorly in the *trans* conformation (Shih et al., 2012; Ahuja et al., 2016; Lippens et al., 2016).

Thus, Pin1 may maintain normal tau functions through the conformational change of pT231-tau, but its deregulation leads to tau-related pathology during AD development. However, Pin1 acts on different phosphorylation sites of tau and has opposite results of tau function. This discrepancy may be due to the different characteristics of the diverse physiological and pathological conditions. Therefore, more evidence is needed to clarify the role of Pin1 in phosphorylated tau and its function.

## Pin1 and App Processing

The extracellular deposition of SPs composed of Aβ peptide derived from APP is another neuropathological hallmark of AD (Hardy and Selkoe, 2002). The human APP gene is located on chromosome 21 and encodes a type I transmembrane protein that plays important roles in neuronal growth, survival, and repair (Thinakaran and Koo, 2008). Upon synthesis in the endoplasmic reticulum, APP undergoes trafficking through the Golgi/trans-Golgi network (TGN) toward the plasma membrane, where it accumulates and internalizes to the endosomes (Selkoe et al., 1996; Thinakaran and Koo, 2008; Pastorino et al., 2012). APP is processed by two different proteolytic processes, the non-amyloidogenic pathway and amyloidogenic pathway (Koo and Squazzo, 1994; Selkoe et al., 1996; Hardy and Selkoe, 2002; Nunan and Small, 2002; Vetrivel and Thinakaran, 2006). In the non-amyloidogenic processing pathway, APP is cleaved by α-secretase at a site within the sequence of Aβ at the plasma membrane, generating soluble extracellular sAPPα with neurotrophic properties and a C-terminal fragment, C83; C83 is further cleaved by γ-secretase to generate the APP intracellular domain (ACID) and a small p3 fragment, thus avoiding Aβ pathology (Esch et al., 1990; Sisodia et al., 1990; Selkoe et al., 1996; Hardy and Selkoe, 2002; Thinakaran and Koo, 2008). In the amyloidogenic processing pathway, APP is internalized to early endosomes through Fe65 and is cleaved by β-secretase to generate soluble sAPPβ and a C-terminal fragment, C99; C99 is further cleaved by γ-secretase in late endosomes to generate the ACID and intact Aβ, inducing Aβ pathology, which is elevated in AD (Selkoe et al., 1996; Wolfe et al., 1999; Yan et al., 1999; Cai et al., 2001; Hardy and Selkoe, 2002; Thinakaran and Koo, 2008).

APP processing and Aβ generation are regulated by the phosphorylation of the intracellular C-terminal fragment (Pastorino and Lu, 2005; Suzuki and Nakaya, 2008). Notably, the phosphorylation of APP at the T668-P motif is increased in the brains of AD patients compared with those of age-matched controls, facilitating the amyloidogenic processing pathway and Aβ generation (Lee et al., 2003). Importantly, Pin1 binds to APP specifically on the phosphorylated T668-P motif *in vitro* and *in vivo* (Pastorino et al., 2006). The binding of Pin1 to phosphorylated T668-P accelerates its isomerization from *cis* to *trans* by over 1,000-fold, as visualized by NMR spectroscopy (Ramelot et al., 2000; Ramelot and Nicholson, 2001; Pastorino et al., 2006). The overexpression of Pin1 significantly decreases Aβ secretion *in vitro*, while Pin1 ablation dramatically increases insoluble Aβ42 secretion in cell models and mouse models in an age-dependent manner (Pastorino et al., 2006). Pin1 is colocalized with APP at the plasma membrane and in clathrin-coated vesicles rather than endosomes, and Pin1 inhibition leads to reduced APP levels at the plasma membrane (Pastorino et al., 2006, 2012). Pin1 influences the levels of Fe65, which can interact with APP and facilitate amyloidogenic APP processing (Pastorino et al., 2012, 2013). Thus, Pin1 isomerizes APP to the *trans* conformation, controls the intracellular localization and internalization of APP, modulates AICD in a Fe65-dependent manner, and thus exerts a protective function against Aβ pathology, indicating that the Pin1-regulated prolyl isomerization of APP plays a key role in regulating Aβ pathology.

**FIGURE 2:**
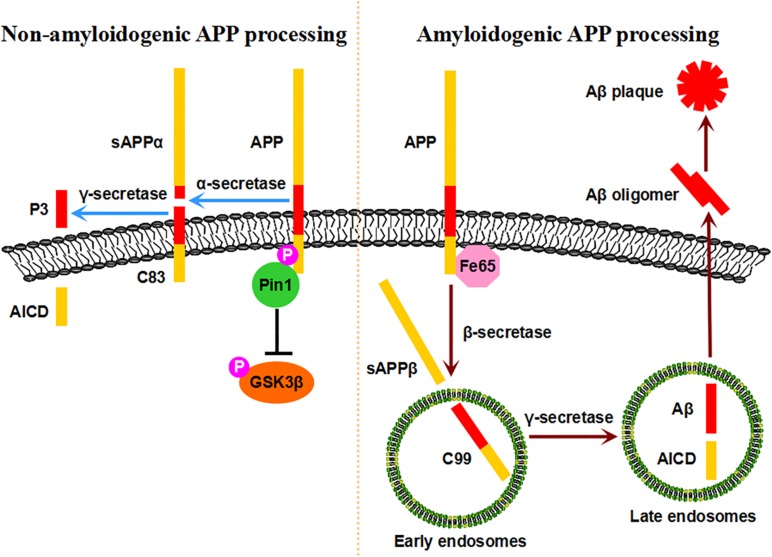
Pin1-mediated APP processing. Pin1 binds to the pT668-P motif of APP, accelerates its isomerization from *cis* to *trans*, stabilizes APP at the plasma membrane, and ultimately promotes the non-amyloidogenic processing pathway. Moreover, conformational changes in APP may affect the binding of Fe65, thus preventing the amyloidogenic processing pathway. Furthermore, Pin1 promotes APP protein turnover by decreasing GSK3β activity. Under physiological conditions, sufficient levels of Pin1 protect healthy neurons against Aβ pathology. When Pin1 expression/activity is inhibited, the amyloidogenic processing pathway is dominant, resulting in Aβ production and plaques.

A number of protein kinases responsible for phosphorylating APP at the T668-P motif are abnormally elevated in the AD brain, such as GSK3β, SAPK1b/JNK3, Cdc2, and Cdk5 (Zhou et al., 2000; Lee et al., 2003, 2011b; Lu and Zhou, 2007; Ma et al., 2012a). Among them, GSK3β is a widely expressed proline-directed serine/threonine kinase that is implicated in a number of physiological processes in the nervous system (Jiang et al., 2005; Yoshimura et al., 2005; Castano et al., 2010). The aberrant regulation of GSK3β contributes to major neurological disorders, including both familial and sporadic AD (Hooper et al., 2008; Peineau et al., 2008). The hyperactivity of GSK3β increases Aβ production, while the inhibition of GSK3β reduces plaques *in vitro* and *in vivo* (Lovestone et al., 1994; Flaherty et al., 2000; Engel et al., 2006; Hurtado et al., 2012). Ma and colleagues showed that Pin1 directly binds to the phosphorylated T330-P motif in GSK3β and inhibits its kinase activity *in vitro* and *in vivo* (Ma et al., 2012a). The suppression of Pin1 causes GSK3β activation, leading to increased levels of T668-phosphorylated APP and amyloidogenic APP processing. In addition, Pin1 promotes APP protein degradation by binding to the phosphorylated T330-P motif of GSK3β (Ma et al., 2012a; Xiong et al., 2013). Thus, Pin1 promotes APP protein turnover by inhibiting GSK3β activity, suggesting a novel neuroprotective role of Pin1 against Aβ pathology. However, contrasting results showed that in the Pin1-deficient mice Aβ was lower and Pin1 promoted Aβ production *in vitro* (Akiyama et al., 2005). The discrepancy might due to the usage of β-cleaved carboxy-terminal frangment C99 instead of full-length APP and detection of the mouse brain at an unspecified age. Thus, in healthy neurons, sufficient levels of Pin1 promote the non-amyloidogenic processing pathway of APP and its turnover, reducing Aβ secretion. However, when Pin1 expression is reduced or its activity is inhibited, amyloidogenic APP processing is increased, resulting in Aβ production and Aβ plaques in the AD brain (Figure 2).

## Pin1 Mouse Models of AD

Animal models recapitulating the characteristics of AD are of vital importance not only for performing *in vivo* studies that explore molecular mechanisms but also for providing preclinical subjects for potential drug candidates. Due to research on familial forms of AD, transgenic mouse models have been developed and widely used (Games et al., 1995; Duff et al., 1996; Hsiao et al., 1996; Lewis et al., 2001; Gotz and Ittner, 2008). However, mutated tau and APP overexpression mice do not recapitulate all features of AD (Drummond and Wisniewski, 2017). Pin1 knockout (KO) mice were initially created to explore the function of Pin1 in mammalian cells, and they were viable and developed normally to adulthood (Fujimori et al., 1999). Importantly, Pin1 KO mice are the first mouse models to show both tau-related and Aβ pathology when a specific gene is deleted (Liou et al., 2003; Pastorino et al., 2006; Lu and Zhou, 2007; Lee et al., 2011b). Pin1 KO mice show tau hyperphosphorylation leading to age-dependent tau filament formation and NFT-like pathologies compared with their wild-type littermates (Liou et al., 2003). Pin1-KO mice also show neuronal loss and progressive age-dependent motor and behavioral deficits, such as abnormal limb-clasping reflexes, hunched posture, reduced mobility, and eye irritation (Liou et al., 2003). When neuron-specific Pin1 transgenic (Tg) mice are bred with wild-type tau Tg mice, Pin1 overexpression reduces tau hyperphosphorylation, NFT specific conformations, and aggregation (Lim et al., 2008). Surprisingly, in Tg mice overexpressing human P301L mutant tau, which causes frontotemporal dementia with parkinsonism linked to chromosome 17 (FTDP-17), Pin1 KO drastically decreases the total tau levels and the hyperphosphorylation of tau, while Pin1-Tg promotes tau hyperphosphorylation, tau aggregation, and NFT conformation (Lim et al., 2008). This unexpected discrepancy was explained that the mutation might somehow render the pT231 motif in tau to be favored in the *trans* conformations (Lim et al., 2008). Therefore, Pin1 overexpression might accelerate the isomerization of the protective *trans* conformation to the pathogenic *cis* conformation, whereas Pin1 inhibition contributes to maintaining the *trans* conformation, facilitating P301L tau degradation (Lim et al., 2008). Since no tau mutations have been found in AD, tau-related pathology induced by exogenous overexpression of mutant tau may be different from human AD in terms of molecular regulatory mechanisms. In addition, when Pin1 KO mice have been bred with tau-Tg mice, these mice exhibit increased *cis* pT231-tau, but decreased *trans* pT231-tau levels, supporting the Pin1-mediated suppression of tau-related neurodegeneration in mice (Nakamura et al., 2012).

Pin1 ablation in mice also affects APP processing in APP-overexpressing mouse brains (Pastorino et al., 2006). Compared with the wild-type littermates, Pin1 KO mice exhibit increased levels of insoluble Aβ42, the major toxic species, at 15 months of age, but not at 6 months of age, suggesting that Pin1 KO promotes amyloidogenic APP processing in an age-dependent manner (Pastorino et al., 2006). Pin1 ablation in APP-Tg2576 mice significantly induces insoluble Aβ42 species and increases soluble APPβ levels at 6 months of age. These Aβ42 species are mainly localized to multivesicular bodies of neurons that experience Aβ plaque pathology (Pastorino et al., 2006). Thus, Pin1 is a unique protein, the deletion of which causes age-dependent tau-related and Aβ pathologies, suggesting evidence of a molecular link between tangles and plagues and a protective role of Pin1 against AD.

## Pin1 in Diagnostic and Therapeutic Strategies for AD

Following a series of abnormal tau hyperphosphorylation that induces the formation of NFTs, pT231 appears to be the first detectable phosphorylation site of tau (Luna-Munoz et al., 2007). Due to the elevated sequestration of pT231-tau into the tangles and the decreased levels of pT231-tau that enter the cerebrospinal fluid (CSF), pT231-tau provides an early and specific biomarker of AD progression (Hampel et al., 2001; Spiegel et al., 2016). The assessment of pT231-tau in the CSF has been regarded as a good predictor of conversion from MCI to AD (Ewers et al., 2007). However, the presence of individual variations using pT231-tau might impede its application as a standardized test, whereas whether the existence of distinct forms (*cis* or *trans*) of pT231-tau helps to explain the variations remains to be investigated (Nakamura et al., 2012). Notably, *cis* and *trans* pT231-tau forms, which are regulated by Pin1, can be distinguished by recently developed conformation-specific antibodies (Nakamura et al., 2012; Kondo et al., 2015). Importantly, *cis* pT231-tau appears early in MCI, is pathologically more relevant, and contributes to AD (Nakamura et al., 2012). Current diagnostic approaches using CSF or positron emission tomography (PET) are either invasive or expensive, making it difficult to achieve early diagnosis using these approaches (Wang et al., 2017; Long and Holtzman, 2019). Commonly known markers used as diagnostic methods are often detectable months or years after the initiation of AD pathogenesis. Therefore, early detectable concentrations of *cis* pT231-tau, changes in *cis* pT231-tau levels, and the ratio of *cis* pT231-tau to *trans* pT231-tau in body fluids and blood from normal and AD patients might be better and more standardized biomarkers for early diagnosis.

To date, AD remains incurable, and a pool of issues remains to be solved. First and foremost, whether Aβ pathology occurs first and induces tau-related pathology, or vice versa, is still controversial. The answer to this question may influence the efficacy of targeted therapies specific for Aβ or tau. Moreover, available therapeutic strategies primarily focus on slowing down the progression of cognitive decline and neurodegeneration rather than targeting essential pathways (Pastorino et al., 2013). Furthermore, the administration of drugs at late stages due to the lack of early diagnosis may dramatically attenuate the efficacy since AD usually takes more than a decade to develop. Notably, the discovery of the Pin1-catalyzed *cis/trans* isomerization of phosphorylated S/T-P motifs in tau and APP and *cis* pT231-tau, but not *trans* pT231-tau, as an early and potent driver in MCI and AD, offers an attractive and promising therapeutic strategy for AD. A generation of mouse monoclonal antibodies specific for *cis* pT231-tau has been developed and shown to eliminate pathologic *cis* pT231-tau and prevent tau-related pathology development and spread (Kondo et al., 2015; Albayram et al., 2017). Importantly, immunotherapy employing this strategy specifically aims at the earliest possible pathogenic form of tau rather than the physiological *trans* form of pT231-tau with normal functions in AD (Kondo et al., 2015). Thus, further humanization of the *cis* pT231-tau antibody is conducive to developing novel therapeutic strategies for AD.

Because Pin1 plays an important role in preventing tau-related and Aβ pathologies in AD, the upregulation and/or activation of Pin1 could be a viable strategy for AD treatment. However, Pin1 overexpression contributes to a number of cancers, eliminating the possibility of direct administration (Zhou and Lu, 2016). Aberrant Pin1 elevation has been shown to be involved in many signaling events such as cell cycle coordination, chromosome instability, proliferation, migration, metastasis, and apoptosis in cancer (Zhou and Lu, 2016). Indeed, Pin1 is known to activate 56 oncogenes and inactivate 26 tumor suppressors by regulating their activity, protein interaction, stability, and cellular localization (Cheng and Tse, 2019; Yu et al., 2020). Pin1 overexpression in mammary gland induces chromosome instability and leads to breast cancer development and Pin1 ablation effectively prevents tumorigenesis by overexpressing Neu in animal models (Wulf et al., 2004; Suizu et al., 2006). Therefore, direct Pin1 activation in brain might cause malignant brain tumor. If we could specifically deliver Pin1 activator to neurons, it might be useful because neurons do not divide or proliferate. In addition, therapeutic strategies targeting Pin1 also focus on the upstream regulators of Pin1 such as DAPK1 or targets such as *cis* or *trans* pT231-tau. Indeed, the inhibition of DAPK1 has been shown to attenuate tau hyperphosphorylation and Aβ production (Kim et al., 2014, 2016, 2019; You et al., 2017; Chen et al., 2019). Thus, Pin1-related therapeutic strategies might be valuable in the treatment of AD.

## Conclusion

The peptidyl-prolyl *cis/trans* isomerase Pin1 is a crucial regulator that is implicated in a wide variety of physiological and pathological activities. The deregulation of Pin1 expression and/or activity is associated with the development of cancer and neurodegeneration, including AD. Interestingly, Pin1 regulates the conformational change of both tau and APP and has protective effects against tau-related and Aβ pathology, suggesting that Pin1 might be a novel and promising candidate for exploring the molecular mechanisms, diagnosis, and treatment of AD.

The availability of drug candidates largely depends on animal models. Currently, many types of single or biogenic Tg mice are broadly used to study tau-related and Aβ pathologies; however, these mice fail to recapitulate all aspects of human AD. Pin1 KO mice develop both tau-related and Aβ pathologies in an age-dependent manner by employing endogenous tau and APP proteins, providing an attractive *in vivo* model for AD research and drug testing. Novel therapeutic strategies such as *cis* pT231-tau antibodies that target conformation-specific phosphorylated tau or small molecules such as DAPK1 inhibitors might provide effective treatment for human AD. However, many questions, including how to regulate Pin1 levels due to its dual roles in cancer and AD, how to overcome the blood-brain barrier for antibody treatment, and how to validate a suitable time for drug administration in the early stage of AD, remain to be answered before clinical validation. Moreover, Pin1 has been shown to lead different direction of tau phosphorylation and APP processing depending on cellular context. More research is urgently needed to illuminate the underlying roles of Pin in the molecular regulation, early diagnosis, potential treatment, and possible prevention of AD.

## Author Contributions

This review manuscript was conceptualized by LW and TL. YZ prepared figures. DC professionally edited the manuscript. LW, YZ, and TL wrote the manuscript.

## Conflict of Interest

The authors declare that the research was conducted in the absence of any commercial or financial relationships that could be construed as a potential conflict of interest.

## References

[B1] Ahuja P. Cantrelle F. X. Huvent I. Hanoulle X. Lopez J. Smet C. (2016). Proline conformation in a functional tau fragment. *J. Mol. Biol.* 428 79–91. 10.1016/j.jmb.2015.11.023 26655856

[B2] Akiyama H. Shin R. W. Uchida C. Kitamoto T. Uchida T. (2005). Pin1 promotes production of Alzheimer’s amyloid beta from beta-cleaved amyloid precursor protein. *Biochem. Biophys. Res. Commun.* 336 521–529. 10.1016/j.bbrc.2005.08.130 16139797

[B3] Albayram O. Angeli P. Bernstein E. Baxley S. Gao Z. Lu K. P. (2018). Targeting prion-like cis phosphorylated tau pathology in neurodegenerative diseases. *J. Alzheimers Dis. Parkinsonism* 8:443 10.4172/2161-0460.1000443 PMC6122852 30197831

[B4] Albayram O. Herbert M. K. Kondo A. Tsai C. Y. Baxley S. Lian X. (2016). Function and regulation of tau conformations in the development and treatment of traumatic brain injury and neurodegeneration. *Cell Biosci.* 6:59 10.1186/s13578-016-0124-4 PMC5139118 27980715

[B5] Albayram O. Kondo A. Mannix R. Smith C. Tsai C. Y. Li C. (2017). Cis P-tau is induced in clinical and preclinical brain injury and contributes to post-injury sequelae. *Nat. Commun.* 8:1000 10.1038/s41467-017-01068-4 PMC5645414 29042562

[B6] Alonso A. C. Zaidi T. Grundke-Iqbal I. Iqbal K. (1994). Role of abnormally phosphorylated tau in the breakdown of microtubules in Alzheimer disease. *Proc. Natl. Acad. Sci. U.S.A.* 91 5562–5566. 10.1073/pnas.91.12.5562 8202528 PMC44036

[B7] Alzheimer’s Association, (2019). Alzheimer’s disease facts and figures. *Alzheimers Dementia* 15 321–387.

[B8] Ando K. Dourlen P. Sambo A. V. Bretteville A. Belarbi K. Vingtdeux V. (2013). Tau pathology modulates Pin1 post-translational modifications and may be relevant as biomarker. *Neurobiol. Aging* 34 757–769. 10.1016/j.neurobiolaging.2012.08.004 22926167

[B9] Andreotti A. H. (2003). Native state proline isomerization: an intrinsic molecular switch. *Biochemistry* 42 9515–9524. 10.1021/bi0350710 12911293

[B10] Ballatore C. Lee V. M. Trojanowski J. Q. (2007). Tau-mediated neurodegeneration in Alzheimer’s disease and related disorders. *Nat. Rev. Neurosci.* 8 663–672. 10.1038/nrn2194 17684513

[B11] Behrsin C. D. Bailey M. L. Bateman K. S. Hamilton K. S. Wahl L. M. Brandl C. J. (2007). Functionally important residues in the peptidyl-prolyl isomerase Pin1 revealed by unigenic evolution. *J. Mol. Biol.* 365 1143–1162. 10.1016/j.jmb.2006.10.078 17113106

[B12] Binder L. I. Guillozet-Bongaarts A. L. Garcia-Sierra F. Berry R. W. (2005). Tau, tangles, and Alzheimer’s disease. *Biochim. Biophys. Acta* 1739 216–223. 15615640 10.1016/j.bbadis.2004.08.014

[B13] Blair L. J. Baker J. D. Sabbagh J. J. Dickey C. A. (2015). The emerging role of peptidyl-prolyl isomerase chaperones in tau oligomerization, amyloid processing, and Alzheimer’s disease. *J. Neurochem.* 133 1–13. 10.1111/jnc.13033 25628064 PMC4361273

[B14] Bramblett G. T. Goedert M. Jakes R. Merrick S. E. Trojanowski J. Q. Lee V. M. (1993). Abnormal tau phosphorylation at Ser396 in Alzheimer’s disease recapitulates development and contributes to reduced microtubule binding. *Neuron* 10 1089–1099. 10.1016/0896-6273(93)90057-x 8318230

[B15] Bulbarelli A. Lonati E. Cazzaniga E. Gregori M. Masserini M. (2009). Pin1 affects Tau phosphorylation in response to *Abeta oligomers*. *Mol. Cell Neurosci.* 42 75–80. 10.1016/j.mcn.2009.06.001 19520166

[B16] Butterfield D. A. (2019). Phosphoproteomics of Alzheimer disease brain: Insights into altered brain protein regulation of critical neuronal functions and their contributions to subsequent cognitive loss. *Biochim. Biophys. Acta Mol. Basis Dis.* 1865 2031–2039. 10.1016/j.bbadis.2018.08.035 31167728 PMC6602546

[B17] Butterfield D. A. Poon H. F. St Clair D. Keller J. N. Pierce W. M. Klein J. B. (2006). Redox proteomics identification of oxidatively modified hippocampal proteins in mild cognitive impairment: insights into the development of Alzheimer’s disease. *Neurobiol. Dis.* 22 223–232. 10.1016/j.nbd.2005.11.002 16466929

[B18] Cai H. Wang Y. Mccarthy D. Wen H. Borchelt D. R. Price D. L. (2001). BACE1 is the major beta-secretase for generation of Abeta peptides by neurons. *Nat. Neurosci.* 4 233–234. 10.1038/85064 11224536

[B19] Cao L. Wang F. Ge H. Wu P. C. Qu P. Chen G. H. (2013). PIN1-842G/C and -667T/C polymorphisms are not associated with the susceptibility of Alzheimer’s disease: pooled analysis of epidemiologic studies. *Neurosci. Lett.* 535 100–103. 10.1016/j.neulet.2012.12.026 23274710

[B20] Castano Z. Gordon-Weeks P. R. Kypta R. M. (2010). The neuron-specific isoform of glycogen synthase kinase-3beta is required for axon growth. *J. Neurochem.* 113 117–130. 10.1111/j.1471-4159.2010.06581.x 20067585

[B21] Chen C. H. Li W. Sultana R. You M. H. Kondo A. Shahpasand K. (2015). Pin1 cysteine-113 oxidation inhibits its catalytic activity and cellular function in Alzheimer’s disease. *Neurobiol. Dis.* 76 13–23. 10.1016/j.nbd.2014.12.027 25576397 PMC4423621

[B22] Chen D. Wang L. Lee T. H. (2020). Post-translational Modifications of the Peptidyl-Prolyl Isomerase Pin1. *Front. Cell Dev. Biol.* 8:129 10.3389/fcell.2020.00129 PMC7064559 32195254

[B23] Chen D. Zhou X. Z. Lee T. H. (2019). Death-associated protein kinase 1 as a promising drug target in cancer and Alzheimer’s disease. *Recent Pat. Anticancer Drug Discov.* 14 144–157. 10.2174/1574892814666181218170257 30569876 PMC6751350

[B24] Cheng C. W. Tse E. (2019). Targeting PIN1 as a therapeutic approach for hepatocellular carcinoma. *Front. Cell Dev. Biol.* 7:369 10.3389/fcell.2019.00369 PMC6974617 32010690

[B25] Cohen P. (1982). The role of protein phosphorylation in neural and hormonal control of cellular activity. *Nature* 296 613–620. 10.1038/296613a0 6280056

[B26] Corder E. H. Saunders A. M. Strittmatter W. J. Schmechel D. E. Gaskell P. C. Small G. W. (1993). Gene dose of apolipoprotein E type 4 allele and the risk of Alzheimer’s disease in late onset families. *Science* 261 921–923. 10.1126/science.8346443 8346443

[B27] Cortes-Hernandez P. Dominguez-Ramirez L. (2017). Role of cis-trans proline isomerization in the function of pathogenic enterobacterial periplasmic binding proteins. *PLoS One* 12:e0188935 10.1371/journal.pone.0188935 PMC5708682 29190818

[B28] Dakson A. Yokota O. Esiri M. Bigio E. H. Horan M. Pendleton N. (2011). Granular expression of prolyl-peptidyl isomerase PIN1 is a constant and specific feature of Alzheimer’s disease pathology and is independent of tau. Abeta and TDP-43 pathology. *Acta Neuropathol.* 121 635–649. 10.1007/s00401-011-0798-y 21243369 PMC3122037

[B29] Davies D. C. Horwood N. Isaacs S. L. Mann D. M. (1992). The effect of age and Alzheimer’s disease on pyramidal neuron density in the individual fields of the hippocampal formation. *Acta Neuropathol.* 83 510–517. 10.1007/bf00310028 1621507

[B30] Driver J. A. Beiser A. Au R. Kreger B. E. Splansky G. L. Kurth T. (2012). Inverse association between cancer and Alzheimer’s disease: results from the framingham heart study. *BMJ* 344:e1442 10.1136/bmj.e1442 PMC3647385 22411920

[B31] Drubin D. G. Kirschner M. W. (1986). Tau protein function in living cells. *J. Cell Biol.* 103 2739–2746. 10.1083/jcb.103.6.2739 3098742 PMC2114585

[B32] Drummond E. Wisniewski T. (2017). Alzheimer’s disease: experimental models and reality. *Acta Neuropathol.* 133 155–175. 10.1007/s00401-016-1662-x 28025715 PMC5253109

[B33] Du H. Guo L. Fang F. Chen D. Sosunov A. A. Mckhann G. M. (2008). Cyclophilin D deficiency attenuates mitochondrial and neuronal perturbation and ameliorates learning and memory in Alzheimer’s disease. *Nat. Med.* 14 1097–1105. 10.1038/nm.1868 18806802 PMC2789841

[B34] Du H. Guo L. Wu X. Sosunov A. A. Mckhann G. M. Chen J. X. (2014). Cyclophilin D deficiency rescues Abeta-impaired PKA/CREB signaling and alleviates synaptic degeneration. *Biochim. Biophys. Acta* 1842 2517–2527. 10.1016/j.bbadis.2013.03.004 23507145 PMC3868643

[B35] Duff K. Eckman C. Zehr C. Yu X. Prada C. M. Perez-Tur J. (1996). Increased amyloid-beta42(43) in brains of mice expressing mutant presenilin 1. *Nature* 383 710–713. 10.1038/383710a0 8878479

[B36] Eichner T. Kutter S. Labeikovsky W. Buosi V. Kern D. (2016). Molecular mechanism of pin1-tau recognition and catalysis. *J. Mol. Biol.* 428 1760–1775. 10.1016/j.jmb.2016.03.009 26996941

[B37] Engel T. Hernandez F. Avila J. Lucas J. J. (2006). Full reversal of Alzheimer’s disease-like phenotype in a mouse model with conditional overexpression of glycogen synthase kinase-3. *J. Neurosci.* 26 5083–5090. 10.1523/JNEUROSCI.0604-06.2006 16687499 PMC6674262

[B38] Esch F. S. Keim P. S. Beattie E. C. Blacher R. W. Culwell A. R. Oltersdorf T. (1990). Cleavage of amyloid beta peptide during constitutive processing of its precursor. *Science* 248 1122–1124. 10.1126/science.2111583 2111583

[B39] Ewers M. Buerger K. Teipel S. J. Scheltens P. Schroder J. Zinkowski R. P. (2007). Multicenter assessment of CSF-phosphorylated tau for the prediction of conversion of MCI. *Neurology* 69 2205–2212. 10.1212/01.wnl.0000286944.22262.ff 18071141

[B40] Fischer G. Aumuller T. (2003). Regulation of peptide bond cis/trans isomerization by enzyme catalysis and its implication in physiological processes. *Rev. Physiol. Biochem. Pharmacol.* 148 105–150. 10.1007/s10254-003-0011-3 12698322

[B41] Flaherty D. B. Soria J. P. Tomasiewicz H. G. Wood J. G. (2000). Phosphorylation of human tau protein by microtubule-associated kinases: GSK3beta and cdk5 are key participants. *J. Neurosci. Res.* 62 463–472. 10.1002/1097-4547(20001101)62:3&lt;463::AID-JNR16&gt;3.0.CO;2-7 11054815

[B42] Fujimori F. Takahashi K. Uchida C. Uchida T. (1999). Mice lacking Pin1 develop normally, but are defective in entering cell cycle from G(0) arrest. *Biochem. Biophys. Res. Commun.* 265 658–663. 10.1006/bbrc.1999.1736 10600477

[B43] Games D. Adams D. Alessandrini R. Barbour R. Berthelette P. Blackwell C. (1995). Alzheimer-type neuropathology in transgenic mice overexpressing V717F beta-amyloid precursor protein. *Nature* 373 523–527. 10.1038/373523a0 7845465

[B44] Geschwind D. H. (2003). Tau phosphorylation, tangles, and neurodegeneration: the chicken or the egg? *Neuron* 40 457–460. 10.1016/s0896-6273(03)00681-0 14642270

[B45] Giustiniani J. Chambraud B. Sardin E. Dounane O. Guillemeau K. Nakatani H. (2014). Immunophilin FKBP52 induces Tau-P301L filamentous assembly in vitro and modulates its activity in a model of tauopathy. *Proc. Natl. Acad. Sci. U.S.A.* 111 4584–4589. 10.1073/pnas.1402645111 24623856 PMC3970538

[B46] Giustiniani J. Guillemeau K. Dounane O. Sardin E. Huvent I. Schmitt A. (2015). The FK506-binding protein FKBP52 in vitro induces aggregation of truncated Tau forms with prion-like behavior. *FASEB J.* 29 3171–3181. 10.1096/fj.14-268243 25888602

[B47] Giustiniani J. Sineus M. Sardin E. Dounane O. Panchal M. Sazdovitch V. (2012). Decrease of the immunophilin FKBP52 accumulation in human brains of Alzheimer’s disease and FTDP-17. *J. Alzheimers Dis.* 29 471–483. 10.3233/JAD-2011-111895 22233767

[B48] Goedert M. Spillantini M. G. (2006). A century of Alzheimer’s disease. *Science* 314 777–781. 17082447 10.1126/science.1132814

[B49] Goedert M. Spillantini M. G. Cairns N. J. Crowther R. A. (1992). Tau proteins of Alzheimer paired helical filaments: abnormal phosphorylation of all six brain isoforms. *Neuron* 8 159–168. 10.1016/0896-6273(92)90117-v 1530909

[B50] Gotz J. Ittner L. M. (2008). Animal models of Alzheimer’s disease and frontotemporal dementia. *Nat. Rev. Neurosci.* 9 532–544. 10.1042/AN20090042 18568014

[B51] Guo L. Du H. Yan S. Wu X. Mckhann G. M. Chen J. X. (2013). Cyclophilin D deficiency rescues axonal mitochondrial transport in Alzheimer’s neurons. *PLoS One* 8:e54914 10.1371/journal.pone.0054914 PMC3561411 23382999

[B52] Halliwell B. (2006). Oxidative stress and neurodegeneration: where are we now? *J. Neurochem.* 97 1634–1658. 10.1111/j.1471-4159.2006.03907.x 16805774

[B53] Hampel H. Buerger K. Kohnken R. Teipel S. J. Zinkowski R. Moeller H. J. (2001). Tracking of Alzheimer’s disease progression with cerebrospinal fluid tau protein phosphorylated at threonine 231. *Ann. Neurol.* 49 545–546. 11310639

[B54] Han C. H. Lu J. Wei Q. Bondy M. L. Brewster A. M. Yu T. K. (2010). The functional promoter polymorphism (-842G>C) in the PIN1 gene is associated with decreased risk of breast cancer in non-Hispanic white women 55 years and younger. *Breast Cancer Res. Treat* 122 243–249. 10.1007/s10549-009-0682-9 20033770 PMC2883663

[B55] Hanes S. D. Shank P. R. Bostian K. A. (1989). Sequence and mutational analysis of ESS1, a gene essential for growth in Saccharomyces cerevisiae. *Yeast* 5 55–72. 10.1002/yea.320050108 2648698

[B56] Hardy J. Selkoe D. J. (2002). The amyloid hypothesis of Alzheimer’s disease: progress and problems on the road to therapeutics. *Science* 297 353–356. 10.1126/science.1072994 12130773

[B57] Holzer M. Gartner U. Stobe A. Hartig W. Gruschka H. Bruckner M. K. (2002). Inverse association of Pin1 and tau accumulation in Alzheimer’s disease hippocampus. *Acta Neuropathol.* 104 471–481. 10.1007/s00401-002-0581-1 12410395

[B58] Hooper C. Killick R. Lovestone S. (2008). The GSK3 hypothesis of Alzheimer’s disease. *J. Neurochem.* 104 1433–1439. 10.1111/j.1471-4159.2007.05194.x 18088381 PMC3073119

[B59] Hsiao K. Chapman P. Nilsen S. Eckman C. Harigaya Y. Younkin S. (1996). Correlative memory deficits, Abeta elevation, and amyloid plaques in transgenic mice. *Science* 274 99–102. 10.1126/science.274.5284.99 8810256

[B60] Huq A. J. Fransquet P. Laws S. M. Ryan J. Sebra R. Masters C. L. (2019). Genetic resilience to Alzheimer’s disease in APOE epsilon4 homozygotes: a systematic review. *Alzheimers Dement* 15 1612–1623. 10.1016/j.jalz.2019.05.011 31506248

[B61] Hurtado D. E. Molina-Porcel L. Carroll J. C. Macdonald C. Aboagye A. K. Trojanowski J. Q. (2012). Selectively silencing GSK-3 isoforms reduces plaques and tangles in mouse models of Alzheimer’s disease. *J. Neurosci.* 32 7392–7402. 10.1523/JNEUROSCI.0889-12.2012 22623685 PMC3368584

[B62] Iqbal K. Liu F. Gong C. X. (2016). Tau and neurodegenerative disease: the story so far. *Nat. Rev. Neurol.* 12 15–27. 10.1038/nrneurol.2015.225 26635213

[B63] Iqbal K. Liu F. Gong C. X. Alonso Adel C. Grundke-Iqbal I. (2009). Mechanisms of tau-induced neurodegeneration. *Acta Neuropathol.* 118 53–69. 19184068 10.1007/s00401-009-0486-3 PMC2872491

[B64] Ittner L. M. Gotz J. (2011). Amyloid-beta and tau–a toxic pas de deux in Alzheimer’s disease. *Nat. Rev. Neurosci.* 12 65–72. 10.1038/nrn2967 21193853

[B65] Jiang H. Guo W. Liang X. Rao Y. (2005). Both the establishment and the maintenance of neuronal polarity require active mechanisms: critical roles of GSK-3beta and its upstream regulators. *Cell* 120 123–135. 10.1016/j.cell.2004.12.033 15652487

[B66] Kim B. M. You M. H. Chen C. H. Lee S. Hong Y. Hong Y. (2014). Death-associated protein kinase 1 has a critical role in aberrant tau protein regulation and function. *Cell Death Dis.* 5:e1237 10.1038/cddis.2014.216 PMC4047864 24853415

[B67] Kim B. M. You M. H. Chen C. H. Suh J. Tanzi R. E. Ho Lee T. (2016). Inhibition of death-associated protein kinase 1 attenuates the phosphorylation and amyloidogenic processing of amyloid precursor protein. *Hum. Mol. Genet.* 25 2498–2513. 10.1093/hmg/ddw114 27094130 PMC6086563

[B68] Kim N. Chen D. Zhou X. Z. Lee T. H. (2019). Death-associated protein kinase 1 phosphorylation in neuronal cell death and Neurodegenerative disease. *Int. J. Mol. Sci.* 20:3131 10.3390/ijms20133131 PMC6651373 31248062

[B69] Kimura T. Tsutsumi K. Taoka M. Saito T. Masuda-Suzukake M. Ishiguro K. (2013). Isomerase Pin1 stimulates dephosphorylation of tau protein at cyclin-dependent kinase (Cdk5)-dependent Alzheimer phosphorylation sites. *J. Biol. Chem.* 288 7968–7977. 10.1074/jbc.M112.433326 23362255 PMC3597833

[B70] Kondo A. Shahpasand K. Mannix R. Qiu J. Moncaster J. Chen C. H. (2015). Antibody against early driver of neurodegeneration cis P-tau blocks brain injury and tauopathy. *Nature* 523 431–436. 10.1038/nature14658 26176913 PMC4718588

[B71] Koo E. H. Squazzo S. L. (1994). Evidence that production and release of amyloid beta-protein involves the endocytic pathway. *J. Biol. Chem.* 269 17386–17389. 8021238

[B72] Kutter S. Eichner T. Deaconescu A. M. Kern D. (2016). Regulation of microtubule assembly by Tau and not by Pin1. *J. Mol. Biol.* 428 1742–1759. 10.1016/j.jmb.2016.03.010 26996940

[B73] Lambert J. C. Bensemain F. Chapuis J. Cottel D. Amouyel P. (2006). Association study of the PIN1 gene with Alzheimer’s disease. *Neurosci. Lett.* 402 259–261. 10.1016/j.neulet.2006.04.010 16701948

[B74] Landrieu I. Leroy A. Smet-Nocca C. Huvent I. Amniai L. Hamdane M. (2010). NMR spectroscopy of the neuronal tau protein: normal function and implication in Alzheimer’s disease. *Biochem. Soc. Trans.* 38 1006–1011. 10.1042/BST0381006 20658994

[B75] Landrieu I. Smet C. Wieruszeski J. M. Sambo A. V. Wintjens R. Buee L. (2006). Exploring the molecular function of PIN1 by nuclear magnetic resonance. *Curr. Protein Pept. Sci.* 7 179–194. 10.2174/138920306777452303 16787258

[B76] Landrieu I. Smet-Nocca C. Amniai L. Louis J. V. Wieruszeski J. M. Goris J. (2011). Molecular implication of PP2A and Pin1 in the Alzheimer’s disease specific hyperphosphorylation of Tau. *PLoS One* 6:e21521 10.1371/journal.pone.0021521 PMC3121875 21731772

[B77] Lane C. A. Hardy J. Schott J. M. (2018). Alzheimer’s disease. *Eur. J. Neurol.* 25 59–70. 28872215 10.1111/ene.13439

[B78] Lanke V. Moolamalla S. T. R. Roy D. Vinod P. K. (2018). Integrative analysis of hippocampus gene expression profiles identifies network alterations in aging and Alzheimer’s disease. *Front. Aging Neurosci.* 10:153 10.3389/fnagi.2018.00153 PMC5974201 29875655

[B79] Lee G. Neve R. L. Kosik K. S. (1989). The microtubule binding domain of tau protein. *Neuron* 2 1615–1624. 10.3390/ijms20030487 2516729

[B80] Lee M. S. Kao S. C. Lemere C. A. Xia W. Tseng H. C. Zhou Y. (2003). APP processing is regulated by cytoplasmic phosphorylation. *J. Cell Biol.* 163 83–95. 10.1083/jcb.200301115 14557249 PMC2173445

[B81] Lee T. H. Chen C. H. Suizu F. Huang P. Schiene-Fischer C. Daum S. (2011a). Death-associated protein kinase 1 phosphorylates Pin1 and inhibits its prolyl isomerase activity and cellular function. *Mol. Cell* 42 147–159. 10.1016/j.molcel.2011.03.005 21497122 PMC3088080

[B82] Lee T. H. Pastorino L. Lu K. P. (2011b). Peptidyl-prolyl cis-trans isomerase Pin1 in ageing, cancer and Alzheimer disease. *Expert Rev. Mol. Med.* 13:e21 10.1017/S1462399411001906 21682951

[B83] Lee V. M. Balin B. J. Otvos L. Jr. Trojanowski J. Q. (1991). A68: a major subunit of paired helical filaments and derivatized forms of normal Tau. *Science* 251 675–678. 10.1126/science.1899488 1899488

[B84] Lee Y. M. Liou Y. C. (2018). Gears-In-Motion: The Interplay of WW and PPIase Domains in Pin1. *Front. Oncol.* 8:469 10.3389/fonc.2018.00469 PMC6232885 30460195

[B85] Lewis J. Dickson D. W. Lin W. L. Chisholm L. Corral A. Jones G. (2001). Enhanced neurofibrillary degeneration in transgenic mice expressing mutant tau and APP. *Science* 293 1487–1491. 10.1126/science.1058189 11520987

[B86] Lim J. Balastik M. Lee T. H. Nakamura K. Liou Y. C. Sun A. (2008). Pin1 has opposite effects on wild-type and P301L tau stability and tauopathy. *J. Clin. Invest.* 118 1877–1889. 10.1172/JCI34308 18431510 PMC2323189

[B87] Liou Y. C. Sun A. Ryo A. Zhou X. Z. Yu Z. X. Huang H. K. (2003). Role of the prolyl isomerase Pin1 in protecting against age-dependent neurodegeneration. *Nature* 424 556–561. 10.1038/nature01832 12891359

[B88] Lippens G. Landrieu I. Smet C. (2007). Molecular mechanisms of the phospho-dependent prolyl cis/trans isomerase Pin1. *FEBS J.* 274 5211–5222. 10.1111/j.1742-4658.2007.06057.x 17892493

[B89] Lippens G. Landrieu I. Smet C. Huvent I. Gandhi N. S. Gigant B. (2016). NMR Meets Tau: Insights into Its Function and Pathology. *Biomolecules* 6:28 10.3390/biom6020028 PMC4919923 27338491

[B90] Long J. M. Holtzman D. M. (2019). Alzheimer disease: an update on pathobiology and treatment strategies. *Cell* 179 312–339. 10.1016/j.cell.2019.09.001 31564456 PMC6778042

[B91] Lovestone S. Reynolds C. H. Latimer D. Davis D. R. Anderton B. H. Gallo J. M. (1994). Alzheimer’s disease-like phosphorylation of the microtubule-associated protein tau by glycogen synthase kinase-3 in transfected mammalian cells. *Curr. Biol.* 4 1077–1086. 10.1016/s0960-9822(00)00246-3 7704571

[B92] Lu J. Yang L. Zhao H. Liu B. Li Y. Wu H. (2011). The polymorphism and haplotypes of PIN1 gene are associated with the risk of lung cancer in Southern and Eastern Chinese populations. *Hum. Mutat.* 32 1299–1308. 10.1002/humu.21574 21850685

[B93] Lu K. P. (2004). Pinning down cell signaling, cancer and Alzheimer’s disease. *Trends Biochem. Sci.* 29 200–209. 10.1016/j.tibs.2004.02.002 15082314

[B94] Lu K. P. Finn G. Lee T. H. Nicholson L. K. (2007). Prolyl cis-trans isomerization as a molecular timer. *Nat. Chem. Biol.* 3 619–629. 10.1038/nchembio.2007.35 17876319

[B95] Lu K. P. Hanes S. D. Hunter T. (1996). A human peptidyl-prolyl isomerase essential for regulation of mitosis. *Nature* 380 544–547. 10.1038/380544a0 8606777

[B96] Lu K. P. Kondo A. Albayram O. Herbert M. K. Liu H. Zhou X. Z. (2016). Potential of the Antibody Against cis-Phosphorylated Tau in the Early Diagnosis, Treatment, and Prevention of Alzheimer Disease and Brain Injury. *JAMA Neurol.* 73 1356–1362. 10.1001/jamaneurol.2016.2027 27654282

[B97] Lu K. P. Liou Y. C. Vincent I. (2003). Proline-directed phosphorylation and isomerization in mitotic regulation and in Alzheimer’s Disease. *Bioessays* 25 174–181. 10.1002/bies.10223 12539244

[B98] Lu K. P. Liou Y. C. Zhou X. Z. (2002). Pinning down proline-directed phosphorylation signaling. *Trends Cell Biol.* 12 164–172. 10.1016/s0962-8924(02)02253-5 11978535

[B99] Lu K. P. Zhou X. Z. (2007). The prolyl isomerase PIN1: a pivotal new twist in phosphorylation signalling and disease. *Nat. Rev. Mol. Cell Biol.* 8 904–916. 10.1038/nrm2261 17878917

[B100] Lu P. J. Wulf G. Zhou X. Z. Davies P. Lu K. P. (1999a). The prolyl isomerase Pin1 restores the function of Alzheimer-associated phosphorylated tau protein. *Nature* 399 784–788. 10.1038/21650 10391244

[B101] Lu P. J. Zhou X. Z. Shen M. Lu K. P. (1999b). Function of WW domains as phosphoserine- or phosphothreonine-binding modules. *Science* 283 1325–1328. 10.1126/science.283.5406.1325 10037602

[B102] Lu Y. Huang G. L. Pu X. X. He Y. X. Li B. B. Liu X. Y. (2013). Association between PIN1 promoter polymorphisms and risk of nasopharyngeal carcinoma. *Mol. Biol. Rep.* 40 3777–3782. 10.1007/s11033-012-2454-6 23269625

[B103] Luna-Munoz J. Chavez-Macias L. Garcia-Sierra F. Mena R. (2007). Earliest stages of tau conformational changes are related to the appearance of a sequence of specific phospho-dependent tau epitopes in Alzheimer’s disease. *J. Alzheimers Dis.* 12 365–375. 10.3233/jad-2007-12410 18198423

[B104] Ma S. L. Pastorino L. Zhou X. Z. Lu K. P. (2012a). Prolyl isomerase Pin1 promotes amyloid precursor protein (APP) turnover by inhibiting glycogen synthase kinase-3beta (GSK3beta) activity: novel mechanism for Pin1 to protect against Alzheimer disease. *J. Biol. Chem.* 287 6969–6973. 10.1074/jbc.C111.298596 22184106 PMC3293570

[B105] Ma S. L. Tang N. L. Tam C. W. Lui V. W. Lam L. C. Chiu H. F. (2012b). A PIN1 polymorphism that prevents its suppression by AP4 associates with delayed onset of Alzheimer’s disease. *Neurobiol. Aging* 33 804–813. 10.1016/j.neurobiolaging.2010.05.018 20580132 PMC2988914

[B106] Markesbery W. R. (1997). Oxidative stress hypothesis in Alzheimer’s disease. *Free Radic Biol. Med.* 23 134–147. 9165306 10.1016/s0891-5849(96)00629-6

[B107] Matsuo E. S. Shin R. W. Billingsley M. L. Van Devoorde A. O’connor M. Trojanowski J. Q. (1994). Biopsy-derived adult human brain tau is phosphorylated at many of the same sites as Alzheimer’s disease paired helical filament tau. *Neuron* 13 989–1002. 10.1016/0896-6273(94)90264-x 7946342

[B108] Mckhann G. M. Knopman D. S. Chertkow H. Hyman B. T. Jack C. R. Jr. Kawas C. H. (2011). The diagnosis of dementia due to Alzheimer’s disease: recommendations from the National Institute on Aging-Alzheimer’s Association workgroups on diagnostic guidelines for Alzheimer’s disease. *Alzheimers Dement* 7 263–269. 10.1016/j.jalz.2011.03.005 21514250 PMC3312024

[B109] Mu Y. Gage F. H. (2011). Adult hippocampal neurogenesis and its role in Alzheimer’s disease. *Mol. Neurodegener.* 6:85 10.1186/1750-1326-6-85 PMC3261815 22192775

[B110] Nakamura K. Greenwood A. Binder L. Bigio E. H. Denial S. Nicholson L. (2012). Proline isomer-specific antibodies reveal the early pathogenic tau conformation in Alzheimer’s disease. *Cell* 149 232–244. 10.1016/j.cell.2012.02.016 22464332 PMC3601591

[B111] Nelson P. T. Alafuzoff I. Bigio E. H. Bouras C. Braak H. Cairns N. J. (2012). Correlation of Alzheimer disease neuropathologic changes with cognitive status: a review of the literature. *J. Neuropathol. Exp. Neurol.* 71 362–381. 10.1097/NEN.0b013e31825018f7 22487856 PMC3560290

[B112] Nestler E. J. Greengard P. (1983). Protein phosphorylation in the brain. *Nature* 305 583–588. 6312325 10.1038/305583a0

[B113] Nowotny P. Bertelsen S. Hinrichs A. L. Kauwe J. S. Mayo K. Jacquart S. (2007). Association studies between common variants in prolyl isomerase Pin1 and the risk for late-onset Alzheimer’s disease. *Neurosci. Lett.* 419 15–17. 10.1016/j.neulet.2007.03.071 17482359 PMC1952685

[B114] Nunan J. Small D. H. (2002). Proteolytic processing of the amyloid-beta protein precursor of Alzheimer’s disease. *Essays Biochem.* 38 37–49. 10.1042/bse0380037 12463160

[B115] Nunomura A. Perry G. Aliev G. Hirai K. Takeda A. Balraj E. K. (2001). Oxidative damage is the earliest event in Alzheimer disease. *J. Neuropathol. Exp. Neurol.* 60 759–767. 10.1093/jnen/60.8.759 11487050

[B116] Oliveira J. Costa M. De Almeida M. S. C. Da Cruz E. S. O. A. B. Henriques A. G. (2017). Protein phosphorylation is a key mechanism in Alzheimer’s disease. *J. Alzheimers Dis.* 58 953–978. 10.3233/JAD-170176 28527217

[B117] Park J. S. Lee J. Jung E. S. Kim M. H. Kim I. B. Son H. (2019). Brain somatic mutations observed in Alzheimer’s disease associated with aging and dysregulation of tau phosphorylation. *Nat. Commun.* 10:3090 10.1038/s41467-019-11000-7 PMC6626023 31300647

[B118] Pastorino L. Kondo A. Zhou X. Z. Lu K. P. (2013). *Pin1 Protects Against Alzheimer’s Disease: One Goal, Multiple Mechaniisms. Understanding Alzheimer’s Disease.* Available online at: https://www.intechopen.com/books/understanding-alzheimer-s-disease/pin1-protects-against-alzheimer-s-disease-one-goal-multiple-mechanisms (February 27, 2013).

[B119] Pastorino L. Lu K. P. (2005). Phosphorylation of the amyloid precursor protein (APP): is this a mechanism in favor or against Alzheimer’s disease. *Neurosci. Res. Commun.* 35 213–231.

[B120] Pastorino L. Ma S. L. Balastik M. Huang P. Pandya D. Nicholson L. (2012). Alzheimer’s disease-related loss of Pin1 function influences the intracellular localization and the processing of AbetaPP. *J. Alzheimers Dis.* 30 277–297. 10.3233/JAD-2012-111259 22430533

[B121] Pastorino L. Sun A. Lu P. J. Zhou X. Z. Balastik M. Finn G. (2006). The prolyl isomerase Pin1 regulates amyloid precursor protein processing and amyloid-beta production. *Nature* 440 528–534. 10.1038/nature04543 16554819

[B122] Peineau S. Bradley C. Taghibiglou C. Doherty A. Bortolotto Z. A. Wang Y. T. (2008). The role of GSK-3 in synaptic plasticity. *Br. J. Pharmacol.* 153(Suppl. 1) S428–S437. 18311157 10.1038/bjp.2008.2 PMC2268071

[B123] Petrucelli L. Dickson D. Kehoe K. Taylor J. Snyder H. Grover A. (2004). CHIP and Hsp70 regulate tau ubiquitination, degradation and aggregation. *Hum. Mol. Genet.* 13 703–714. 10.1093/hmg/ddh083 14962978

[B124] Poppek D. Keck S. Ermak G. Jung T. Stolzing A. Ullrich O. (2006). Phosphorylation inhibits turnover of the tau protein by the proteasome: influence of RCAN1 and oxidative stress. *Biochem. J.* 400 511–520. 10.1042/BJ20060463 16939415 PMC1698600

[B125] Ramakrishnan P. Dickson D. W. Davies P. (2003). Pin1 colocalization with phosphorylated tau in Alzheimer’s disease and other tauopathies. *Neurobiol. Dis.* 14 251–264. 10.1016/s0969-9961(03)00109-8 14572447

[B126] Ramelot T. A. Gentile L. N. Nicholson L. K. (2000). Transient structure of the amyloid precursor protein cytoplasmic tail indicates preordering of structure for binding to cytosolic factors. *Biochemistry* 39 2714–2725. 10.1021/bi992580m 10704223

[B127] Ramelot T. A. Nicholson L. K. (2001). Phosphorylation-induced structural changes in the amyloid precursor protein cytoplasmic tail detected by NMR. *J. Mol. Biol.* 307 871–884. 10.1006/jmbi.2001.4535 11273707

[B128] Ranganathan R. Lu K. P. Hunter T. Noel J. P. (1997). Structural and functional analysis of the mitotic rotamase Pin1 suggests substrate recognition is phosphorylation dependent. *Cell* 89 875–886. 10.1016/s0092-8674(00)80273-1 9200606

[B129] Roberson E. D. Mucke L. (2006). 100 years and counting: prospects for defeating Alzheimer’s disease. *Science* 314 781–784. 10.1126/science.1132813 17082448 PMC3544944

[B130] Rogals M. J. Greenwood A. I. Kwon J. Lu K. P. Nicholson L. K. (2016). Neighboring phosphoSer-Pro motifs in the undefined domain of IRAK1 impart bivalent advantage for Pin1 binding. *FEBS J.* 283 4528–4548. 10.1111/febs.13943 27790836 PMC5298935

[B131] Rong X. F. Sun Y. N. Liu D. M. Yin H. J. Peng Y. Xu S. F. (2017). The pathological roles of NDRG2 in Alzheimer’s disease, a study using animal models and APPwt-overexpressed cells. *CNS Neurosci. Ther.* 23 667–679. 10.1111/cns.12716 28670853 PMC6492714

[B132] Schutkowski M. Bernhardt A. Zhou X. Z. Shen M. Reimer U. Rahfeld J. U. (1998). Role of phosphorylation in determining the backbone dynamics of the serine/threonine-proline motif and Pin1 substrate recognition. *Biochemistry* 37 5566–5575. 10.1021/bi973060z 9548941

[B133] Segat L. Pontillo A. Annoni G. Trabattoni D. Vergani C. Clerici M. (2007). PIN1 promoter polymorphisms are associated with Alzheimer’s disease. *Neurobiol. Aging* 28 69–74. 10.1016/j.neurobiolaging.2005.11.009 16384626

[B134] Selkoe D. J. Yamazaki T. Citron M. Podlisny M. B. Koo E. H. Teplow D. B. (1996). The role of APP processing and trafficking pathways in the formation of amyloid beta-protein. *Ann. N. Y. Acad. Sci.* 777 57–64. 10.1111/j.1749-6632.1996.tb34401.x 8624127

[B135] Shen M. Stukenberg P. T. Kirschner M. W. Lu K. P. (1998). The essential mitotic peptidyl-prolyl isomerase Pin1 binds and regulates mitosis-specific phosphoproteins. *Genes Dev.* 12 706–720. 10.1101/gad.12.5.706 9499405 PMC316589

[B136] Shih H. H. Tu C. Cao W. Klein A. Ramsey R. Fennell B. J. (2012). An ultra-specific avian antibody to phosphorylated tau protein reveals a unique mechanism for phosphoepitope recognition. *J. Biol. Chem.* 287 44425–44434. 10.1074/jbc.M112.415935 23148212 PMC3531756

[B137] Shimura H. Miura-Shimura Y. Kosik K. S. (2004). Binding of tau to heat shock protein 27 leads to decreased concentration of hyperphosphorylated tau and enhanced cell survival. *J. Biol. Chem.* 279 17957–17962. 10.1074/jbc.M400351200 14963027

[B138] Sisodia S. S. Koo E. H. Beyreuther K. Unterbeck A. Price D. L. (1990). Evidence that beta-amyloid protein in Alzheimer’s disease is not derived by normal processing. *Science* 248 492–495. 10.1126/science.1691865 1691865

[B139] Smet C. Sambo A. V. Wieruszeski J. M. Leroy A. Landrieu I. Buee L. (2004). The peptidyl prolyl cis/trans-isomerase Pin1 recognizes the phospho-Thr212-Pro213 site on Tau. *Biochemistry* 43 2032–2040. 10.1021/bi035479x 14967043

[B140] Smet C. Wieruszeski J. M. Buee L. Landrieu I. Lippens G. (2005). Regulation of Pin1 peptidyl-prolyl cis/trans isomerase activity by its WW binding module on a multi-phosphorylated peptide of Tau protein. *FEBS Lett.* 579 4159–4164. 10.1016/j.febslet.2005.06.048 16024016

[B141] Spiegel J. Pirraglia E. Osorio R. S. Glodzik L. Li Y. Tsui W. (2016). Greater specificity for cerebrospinal fluid P-tau231 over P-tau181 in the differentiation of healthy controls from Alzheimer’s disease. *J. Alzheimers Dis.* 49 93–100. 10.3233/JAD-150167 26444757 PMC4694576

[B142] Stoothoff W. H. Johnson G. V. (2005). Tau phosphorylation: physiological and pathological consequences. *Biochim. Biophys. Acta* 1739 280–297. 10.1016/j.bbadis.2004.06.017 15615646

[B143] Suizu F. Ryo A. Wulf G. Lim J. Lu K. P. (2006). Pin1 regulates centrosome duplication, and its overexpression induces centrosome amplification, chromosome instability, and oncogenesis. *Mol. Cell Biol.* 26 1463–1479. 10.1128/MCB.26.4.1463-1479.2006 16449657 PMC1367188

[B144] Sultana R. Boyd-Kimball D. Poon H. F. Cai J. Pierce W. M. Klein J. B. (2006). Oxidative modification and down-regulation of Pin1 in Alzheimer’s disease hippocampus: a redox proteomics analysis. *Neurobiol. Aging* 27 918–925. 10.1016/j.neurobiolaging.2005.05.005 15950321

[B145] Suzuki T. Nakaya T. (2008). Regulation of amyloid beta-protein precursor by phosphorylation and protein interactions. *J. Biol. Chem.* 283 29633–29637. 10.1074/jbc.R800003200 18650433 PMC2662050

[B146] Thinakaran G. Koo E. H. (2008). Amyloid precursor protein trafficking, processing, and function. *J. Biol. Chem.* 283 29615–29619. 10.1074/jbc.R800019200 18650430 PMC2573065

[B147] Tramutola A. Triani F. Di Domenico F. Barone E. Cai J. Klein J. B. (2018). Poly-ubiquitin profile in Alzheimer disease brain. *Neurobiol. Dis.* 118 129–141. 10.1016/j.nbd.2018.07.006 30003951

[B148] Vetrivel K. S. Thinakaran G. (2006). Amyloidogenic processing of beta-amyloid precursor protein in intracellular compartments. *Neurology* 66 S69–S73. 10.1212/01.wnl.0000192107.17175.39 16432149

[B149] Wang J. Gu B. J. Masters C. L. Wang Y. J. (2017). A systemic view of Alzheimer disease - insights from amyloid-beta metabolism beyond the brain. *Nat. Rev. Neurol.* 13 612–623. 10.1038/nrneurol.2017.147 28960209

[B150] Wedemeyer W. J. Welker E. Scheraga H. A. (2002). Proline cis-trans isomerization and protein folding. *Biochemistry* 41 14637–14644. 10.1021/bi020574b 12475212

[B151] Wijsman E. M. Daw E. W. Yu C. E. Payami H. Steinbart E. J. Nochlin D. (2004). Evidence for a novel late-onset Alzheimer disease locus on chromosome 19p13.2. *Am. J. Hum. Genet.* 75 398–409. 10.1086/423393 15248153 PMC1182019

[B152] Wolfe M. S. Xia W. Ostaszewski B. L. Diehl T. S. Kimberly W. T. Selkoe D. J. (1999). Two transmembrane aspartates in presenilin-1 required for presenilin endoproteolysis and gamma-secretase activity. *Nature* 398 513–517. 10.1038/19077 10206644

[B153] Wulf G. Finn G. Suizu F. Lu K. P. (2005). Phosphorylation-specific prolyl isomerization: is there an underlying theme? *Nat. Cell Biol.* 7 435–441. 10.1038/ncb0505-435 15867923

[B154] Wulf G. Garg P. Liou Y. C. Iglehart D. Lu K. P. (2004). Modeling breast cancer in vivo and ex vivo reveals an essential role of Pin1 in tumorigenesis. *EMBO J.* 23 3397–3407. 10.1038/sj.emboj.7600323 15257284 PMC514501

[B155] Xiong Y. S. Wang D. L. Tan L. Wang X. Chen L. M. Gong C. X. (2013). Inhibition of glycogen synthase kinase-3 reverses tau hyperphosphorylation induced by Pin1 down-regulation. *CNS Neurol. Disord. Drug Targets* 12 436–443. 10.2174/1871527311312030016 23469846

[B156] Xu L. Ren Z. Chow F. E. Tsai R. Liu T. Rizzolio F. (2017). Pathological role of peptidyl-Prolyl isomerase Pin1 in the disruption of synaptic plasticity in Alzheimer’s disease. *Neural. Plast* 2017:3270725 10.1155/2017/3270725 PMC5385220 28458925

[B157] Yaffe M. B. Schutkowski M. Shen M. Zhou X. Z. Stukenberg P. T. Rahfeld J. U. (1997). Sequence-specific and phosphorylation-dependent proline isomerization: a potential mitotic regulatory mechanism. *Science* 278 1957–1960. 10.1126/science.278.5345.1957 9395400

[B158] Yamazaki Y. Zhao N. Caulfield T. R. Liu C. C. Bu G. (2019). Apolipoprotein E and Alzheimer disease: pathobiology and targeting strategies. *Nat. Rev. Neurol.* 15 501–518. 10.1038/s41582-019-0228-7 31367008 PMC7055192

[B159] Yan R. Bienkowski M. J. Shuck M. E. Miao H. Tory M. C. Pauley A. M. (1999). Membrane-anchored aspartyl protease with Alzheimer’s disease beta-secretase activity. *Nature* 402 533–537. 10.1038/990107 10591213

[B160] Yoshimura T. Kawano Y. Arimura N. Kawabata S. Kikuchi A. Kaibuchi K. (2005). GSK-3beta regulates phosphorylation of CRMP-2 and neuronal polarity. *Cell* 120 137–149. 10.1016/j.cell.2004.11.012 15652488

[B161] You M. H. Kim B. M. Chen C. H. Begley M. J. Cantley L. C. Lee T. H. (2017). Death-associated protein kinase 1 phosphorylates NDRG2 and induces neuronal cell death. *Cell Death Differ.* 24 238–250. 10.1038/cdd.2016.114 28141794 PMC5299707

[B162] Yu J. H. Im C. Y. Min S.-H. (2020). Function of PIN1 in cancer development and its inhibitors as cancer therapeutics. *Front. Cell Dev. Biol.* 8:120 10.3389/fcell.2020.00120 PMC7089927 32258027

[B163] Zhou X. Z. Kops O. Werner A. Lu P. J. Shen M. Stoller G. (2000). Pin1-dependent prolyl isomerization regulates dephosphorylation of Cdc25C and tau proteins. *Mol. Cell* 6 873–883. 10.1016/s1097-2765(05)00083-3 11090625

[B164] Zhou X. Z. Lu K. P. (2016). The isomerase PIN1 controls numerous cancer-driving pathways and is a unique drug target. *Nat. Rev. Cancer* 16 463–478. 10.1038/nrc.2016.49 27256007

[B165] Zhou X. Z. Lu P. J. Wulf G. Lu K. P. (1999). Phosphorylation-dependent prolyl isomerization: a novel signaling regulatory mechanism. *Cell Mol. Life Sci.* 56 788–806. 10.1007/s000180050026 11212339 PMC11147038

